# Survival and Success Rates of Monolithic Zirconia Restorations Supported by Teeth and Implants in Bruxer versus Non-Bruxer Patients: A Retrospective Study

**DOI:** 10.3390/ma15030833

**Published:** 2022-01-22

**Authors:** Hadas Heller, David Sreter, Adi Arieli, Ilan Beitlitum, Raphael Pilo, Shifra Levartovsky

**Affiliations:** 1 Department of Oral Rehabilitation, The Maurice and Gabriela Goldschleger School of Dental Medicine, Sackler Faculty of Medicine, Tel Aviv University, Tel Aviv 6997801, Israel; heller.hadas@gmail.com (H.H.); sreterd@gmail.com (D.S.); dr.arieli@gmail.com (A.A.); 2 Department of Periodontology and Dental Implantology, The Maurice and Gabriela Goldschleger School of Dental Medicine, Tel Aviv University, Tel Aviv 6997801, Israel; beilan1612@gmail.com; 3 Department of Oral Biology, The Maurice and Gabriela Goldschleger School of Dental Medicine, Sackler Faculty of Medicine, Tel Aviv University, Tel Aviv 6997801, Israel; rafipilo@gmail.com

**Keywords:** zirconia restoration, veneered and non-veneered, bruxer, tooth, dental implant

## Abstract

The aim of this study was to assess retrospectively the survival and success rates of monolithic zirconia restorations supported by teeth and implants in bruxer versus non-bruxer patients. Methods: A total of 15 bruxer and 25 non-bruxer patients attended the recall appointment. The bruxer group (mean age of 61.2 ± 13.3 years and follow-up of 58.7 ± 16.8 months) were treated with 331 monolithic zirconia restorations, while the non-bruxer group, with a comparable mean age and follow-up time, were treated with 306 monolithic zirconia restorations. Clinical data were retrieved from the patients’ files. At the recall appointment, all supporting teeth and implants were examined for biological and technical complications, and the restorations were evaluated using modified California Dental Association (CDA) criteria. Data were statistically analyzed using survival analysis methods. A significance level of *p* < 0.05 was used. A total of 31 versus 27 biologic and technical complications were recorded in the bruxer and non-bruxer groups, respectively. No significant differences were found between the two groups regarding overall complications and survival rate. Regarding the type of complication, a significantly higher rate of veneered porcelain chipping (*p* = 0.045) was observed in the bruxer group. With regard to biological complications, the only complications that exhibited a borderline, although not significant, difference were three fractured teeth exclusively in the bruxer group (*p* = 0.051), which were replaced with implant-supported restorations. Within the limitations of this study, we conclude that there were no significant differences in the overall survival and success rates of the monolithic zirconia restorations in bruxer versus non-bruxer patients, although veneered zirconia restorations and single tooth abutments exhibited a higher rate of complications in the bruxer group.

## 1. Introduction

According to the international consensus obtained in 2013, bruxism, either during sleep or while awake, is defined as a repetitive masticatory muscle activity, characterized by clenching or grinding of the teeth and/or by bracing or thrusting of the mandible [1].

Bruxism has been reported to have a positive association with various biological and technical complications, such as porcelain chipping, tooth cracks/fracture, loosened screws on implants, and abutment/implant fracture. At first, bruxism was considered a contraindication for implant treatment, and since an excessive overload may result in bone loss or even in implant failure, practical guidelines and recommendations were established to minimize implant overload [2]. Recent studies that evaluated the influence of bruxism on implant-supported restorations and implant failure rates have reported contradictory results. Manfredini et al. suggested that bruxism should be considered a risk factor for mechanical rather than biological complications of dental implants [3]. Similarly, Chrcanovic et al. reported higher rates of implant failure and more mechanical and technical complications of implant-supported restorations in bruxers versus a matched group of non-bruxers [4]. In contrast, a recent retrospective study demonstrated a 100% success and survival rate of all implants in bruxer patients, after a mean observation period of 28.2 (±16.8) months [5].

With the development of monolithic all-ceramic materials produced by CAD/CAM technologies, high strength monolithic zirconia restorations have become the treatment of choice for bruxer patients. This is due to the high flexural strength (900–1200 MPa) and fracture toughness (9–10 MPa m0.5) of the monolithic zirconia polycrystals (Y-TZP) [6]. Veneered feldspathic ceramic is sometimes added on the nonfunctioning facial surfaces in order to improve the esthetics of the monolithic zirconia restorations in the anterior zone.

Hansen et al., who assessed the clinical outcomes of monolithic zirconia crowns in the aesthetic zone in 13 patients with severe tooth wear, found that 94% of the crowns had no biological complications, and technical complications were present in only two patients [7]. A recent study that investigated the complication rate of zirconia restorations in 45 patients, including those with clinical signs of bruxism, reported that 80% of catastrophic failures and 76.9% of all complications occurred in patients with clinical signs of bruxism [8]. Despite these values, there were no statistically significant differences in the restoration survival rate in patients with or without clinical signs of bruxism. Hammoudi et al. evaluated the survival rate of monolithic zirconia restorations in patients with extensive tooth wear over an observation period of up to six years, where some of the patients self-reported sleep/day bruxism [9]. These authors reported an overall success rate of 99.7% for the monolithic zirconia restorations. Similarly, Levartovsky et al., who evaluated the clinical performance of teeth- and implant-supported veneered and non-veneered zirconia restorations in patients diagnosed with bruxism, demonstrated an overall mean survival and success rate of 99.6% for all restorations with only minor technical complications [5].

A recent overview of systematic reviews on zirconia-based tooth and implant-supported restorations reported a 5-year satisfactory result with the predominant technical problem being veneering fractures [10]. However, the authors noted that patients with bruxism were examined only sporadically and that most of the studies to evaluate the survival and success of ceramic restorations have excluded bruxer patients as bruxism is considered a potential cause of restoration fracture [11,12]. For this reason, literature data comparing the fate of restorations between bruxer and non- bruxer patients are limited.

The aim of the current study was to assess retrospectively the survival and success rates of monolithic zirconia restorations supported by teeth and implants in bruxer versus non-bruxer patients. The null hypothesis was that there are no significant differences between the two groups.

## 2. Experimental Section

### 2.1. Patients

Prior to the data collection, the study was approved by the Ethics Committee of Tel Aviv University (# 0002485-1), Tel-Aviv, Israel. Written and oral informed consent was provided by all the participants.

The electronic records of patients who had been rehabilitated with monolithic zirconia restorations on teeth or implants in the graduate program of prosthodontics in the Dental Medicine School and in the private practice of the head of the program, who is an experienced prosthodontist (SL), were collected. All demographic, clinical patient-related, and postoperative follow-up data were collected from the patients’ dental records. In addition, an invitation for a recall appointment was sent to these patients together with information about participation in the current study and its purpose.

### 2.2. Inclusion Criteria

Eligible patients were required to have been rehabilitated by veneered and non-veneered monolithic zirconia restorations (Prettau, Zirkonzahn, Israel) on teeth or implants with either single abutments or multiunit fixed partial dentures; to have complete preoperative, intraoperative, and immediate postoperative clinical and radiographic records; and to have a minimum follow-up period of one year postoperatively.

### 2.3. Exclusion Criteria

Exclusion criteria included having less than one year of follow-up after definitive prosthesis insertion; aggressive periodontal disease; an uncontrolled medical condition; incomplete records; and unavailability for recall.

### 2.4. Data Collection

Patients were divided into bruxer and non-bruxer patients at the time of arrival. The grade of bruxism was defined according to the international consensus published by Lobbezoo et al. [1], with reference to the signs and symptoms of bruxism published by the American Academy of Sleep Medicine [13]. The diagnostic grading system of “possible”, “probable”, and “definite” sleep or awake bruxism was based on the patient’s self-report of clenching/grinding during sleep or wakefulness, plus inspection as part of the clinical examination.

All patients were rehabilitated by veneered and non-veneered monolithic zirconia restorations (Prettau, Zirkonzahn, Israel) on teeth or implants with the same surgical and prosthetic approach. Two implant systems (Tapered Screw-Vent^®^, Zimmer Biomet, Palm Beach Gardens, FL, USA; and Lance Implant, MIS/Divident, Or-Yehuda, Israel) were used in this clinical study. After achieving implant osseointegration, analogical impressions for the provisional prostheses on teeth and implants were made and occlusion and esthetic were evaluated. The occlusion was set with maximum intercuspation in centric occlusion and with either canine or group function in lateral excursions. After at least two months of function, final restorations were placed, supporting either single abutment on the tooth or implant without cantilevers or short fixed partial dentures (FPDs) replacing only one pontic. FPDs on implants were either screwed or cemented to the implant abutments. Restorations had a non-veneered monolithic zirconia design in the posterior quadrants, while in the anterior quadrants, veneered feldspathic ceramic was added on the nonfunctioning anterior labial surfaces of the monolithic zirconia. Resin-reinforced glass ionomer luting cement (Fuji Plus, GC Corp., Tokyo, Japan) was used for all tooth-supported restorations while temporary cement (Temp-Bond, Kerr, CA, USA) was used for the cemented implant-supported restorations.

Immediately postoperatively, an occlusal guard (hard occlusal stabilization splint) was registered for all bruxer patients. At the recall appointment, clinical and radiographic records evaluating the survival and success of the monolithic zirconia restorations and the abutments (implants and teeth) were prepared. The periapical radiographs of each abutment were taken with the long cone technique and were compared with the radiographs taken at the time of the definitive prosthesis placement.

Two examiners (HH and DS) were trained and their assessment coordinated prior to performing the radiographic and clinical examination of the monolithic restorations and the abutments (implants and teeth) at the recall appointment. The two examiners assessed all records independently; in cases where their ratings differed, they jointly discussed the issue and reached an agreement.

Biological findings, including the periodontal probing depth (PPD) and bleeding on probing (BoP), were recorded for each abutment tooth or implant. In addition, any other biological complications in the teeth, such as secondary caries, root canal treatment, or extraction, were also noted. Survival of the implant was defined as a functional implant without clinical signs of infection at the time of the examination, even if bone resorption was identified radiographically. Peri-implantitis was diagnosed in the presence of bleeding and/or suppuration on gentle probing, probing depths of ≥6 mm, and bone levels ≥3 mm apical of the most coronal portion of the intraosseous part of the implant. This definition is according to the Consensus report of the 2017 World Workshop on periodontal and peri-implant diseases and conditions [14]. An implant was said to fail if it did not survive during the follow-up period [15,16].

The technical assessment of the monolithic zirconia restorations was in accordance with the modified California Dental Association (CDA) quality evaluation system for assessing surface, color, shape, and marginal integrity [5]. Technical complications observed included zirconia fracture, veneered porcelain chipping, occlusal screw loosening, open proximal contact, and retention loss of the restoration. Porcelain chipping was graded as described by Heintze et al. and Anusavice: grade 1 = polishing; grade 2 = repair; and grade 3 = replacement [17,18].

Each biological and technical event was recorded as a complication. Survival of zirconia restoration was defined as a restoration in situ at the time of the examination. Porcelain chipping that could be repaired by polishing (grade 1) was considered a technical complication, while porcelain, zirconia, or tooth fracture that required repair or replacement (grades 2 and 3) was recorded as a failed restoration. The amount and type of the complications were compared between bruxer and non-bruxer patients.

### 2.5. Statistical Methods and Synthesis of Results

The descriptive statistics of the parameters (i.e., age, follow-up time, complications, and survival), as well as the statistical analysis, employed IBM SPSS Statistics version 23.0 (SPSS, Inc. Chicago, IL, USA). Differences between survival as well as the complications rate in the bruxer vs. the non-bruxer groups were determined by Student’s *t*-test. Fisher’s exact test was used to compare the type of complication between the two independent groups. The survival and complication rates were also compared using the Kaplan–Meier survival analysis method. The degree of statistical significance was considered as *p* < 0.05.

## 3. Results

Among the 50 patients whose dental records were examined, 20 patients were identified as bruxers, and 30 as non-bruxers. A total of 15 bruxers and 25 non-bruxers agreed to participate in the study and were examined at the recall appointment. The 15 bruxer patients (6 females and 9 males) were between 35 and 68 years of age (mean: 61.2 ± 13.3 years) and were followed-up for a mean of 58.7 ± 16.8 months. The 25 non-bruxer patients (15 females and 10 males) were between 56 and 78 years of age (mean: 66.7 ± 7 years) and were followed-up for a mean of 54.1 ± 12.5 months. Details regarding the number of restorations (veneered and non-veneered) and abutments (implants and teeth) for both groups are presented in Table 1.

The two groups were comparable with respect to age and follow-up time (Table 2).

The inter-examiner (HH and DS) reliability analysis (ICC) was 0.88, indicating a high consistency between the two examiners.

In the non-bruxer group, the overall cumulative survival rate of the zirconia restorations was considered to be 100%. In contrast, in the bruxer group, three teeth were fractured at the cement–enamel junction. The three fractured teeth were replaced by implants and restored with new zirconia restorations. Therefore, the cumulative survival rate of the zirconia restorations in the bruxer group was 99.2%.

No significant differences were found between the two groups regarding the mean age, follow-up time, overall complications, and survival rate (Table 2).

A total of 31 complications were recorded in the bruxer group versus 27 in the non-bruxer patients, and the results are summarized in Table 3. Among the bruxer group, 12 patients reported the use of an occlusal guard during sleep.

There were no significant differences by Fisher’s exact test in the overall complications and survival rate between the two groups, but there were some differences when comparing the type of complications.

**Figure 1:**
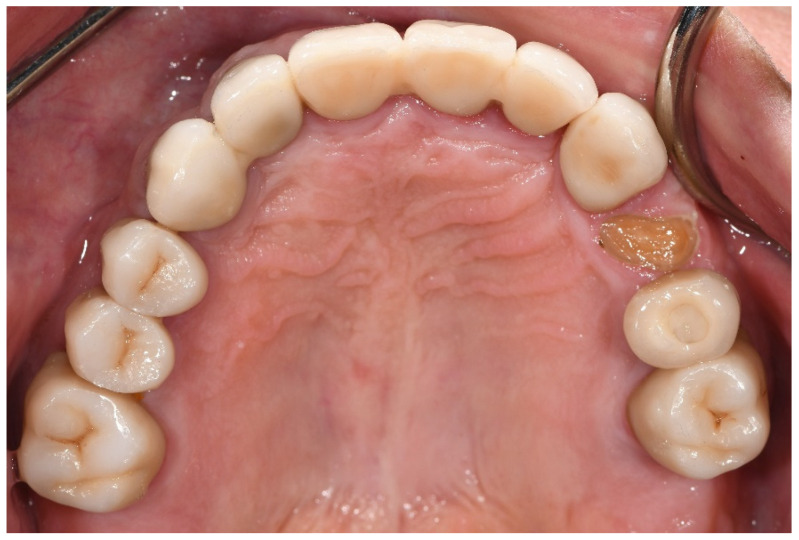
Patient with a fractured upper first pre-molar four years post-cementation. Three years prior, the same patient experienced fracture in an upper second pre-molar, which was replaced with a screw-retained implant restoration.

With regard to biological complications, the only complications that exhibited a borderline, although not significant, difference were three fractured teeth exclusively in the bruxer group (*p* = 0.051). The fractured teeth were in two different patients; one patient with two vital fractured premolars (first tooth after one year of function, the second after four years, Figure 1), and one patient with a non-vital fractured canine (after five years of function). Both patients did not wear an occlusal guard.

The cumulative implant survival rate in the bruxer group was 97.3%, since two implants failed: one failed in a male patient after two years of function due to implant disintegration (Tapered Screw-Vent^®^, Zimmer Biomet) while the other implant failed in a female patient after six years of function due to peri-implantitis (Lance Implant, MIS/Divident). In the non-bruxer group, only one implant failed in a female patient after seven years of function due to peri-implantitis (Lance Implant, MIS/Divident). Therefore, the cumulative implant survival rate in the non-bruxer group was considered as 98.5%. Life table survival analyses concerning implant survival are shown in Table 4 and Table 5 (bruxers and non-bruxers, respectively).

None of the implant failures required replacement of the supported zirconia restoration since they were not single units, but rather part of multiunit fixed partial dentures. Once the implant failed, the zirconia restoration was removed and the implant was pulled out. Subsequently, the inner side of the zirconia crown was filled with composite and the restoration was reattached to the other multiunit abutments.

Secondary caries occurred in 2 of the 269 and in 3 of the 211 abutment teeth of the bruxer and non-bruxer groups, respectively, with no significant difference.

**Figure 2:**
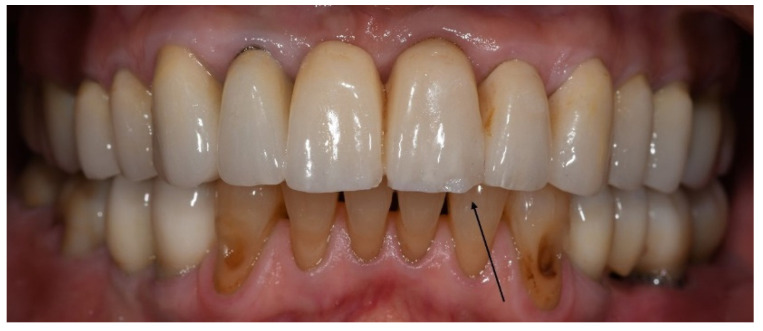
Porcelain chipping on the incisal edge of the veneered monolithic restoration in the upper left central incisor, three years post-cementation. Repair required only polishing.

With regard to technical complications, the rate of porcelain chipping on the incisal edge of the veneered monolithic restorations was significantly higher in the bruxer group than in the non-bruxers (*p* = 0.045). In all cases, porcelain chipping required only polishing (grade 1); therefore, the restorations were not replaced (Figure 2).

Other technical complications, such as screw loosening, minor zirconia fracture, retention loss, and open proximal contacts between implants and teeth, occurred in both groups with no significant differences detected. The minor zirconia fractures required only polishing (grade 1) without the need for restoration replacement. Some of the open proximal contacts observed (five in the bruxers and seven in the non-bruxers) had food impaction and required repair by removing the screwed implant restoration and adding feldspathic porcelain in the contact area. The other open proximal contacts had no food impaction and the patients were only carefully monitored.

**Figure 3:**
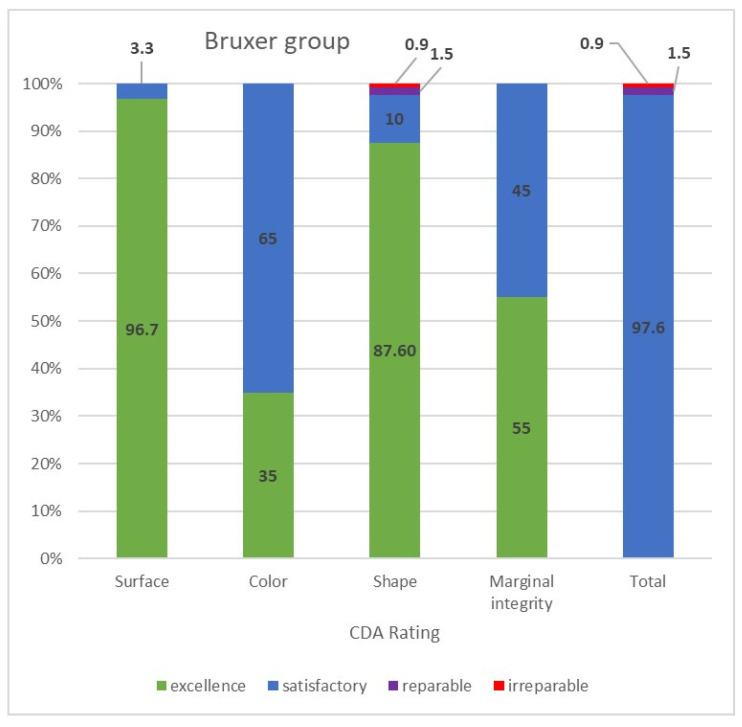
CDA ratings for the bruxer group.

**Figure 4:**
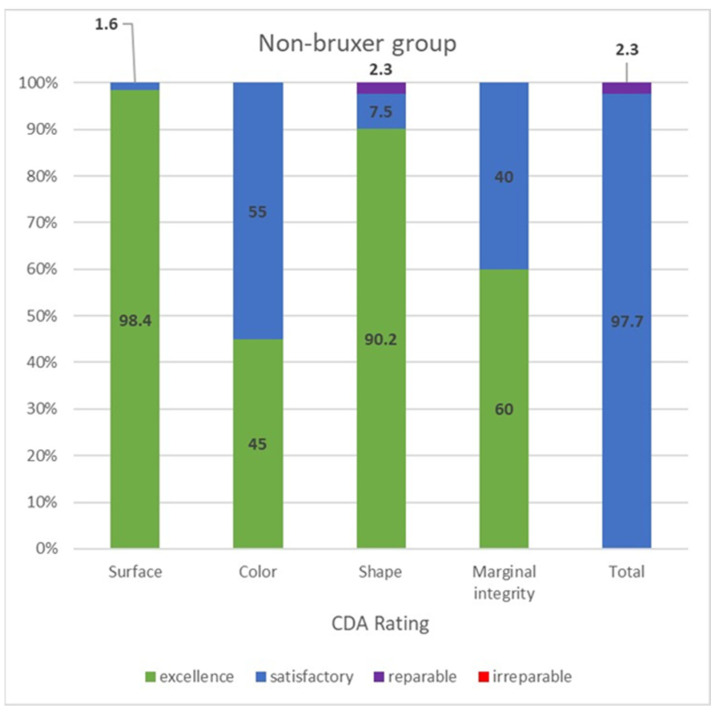
CDA ratings for the non-bruxer group.

The CDA ratings were divided into those of the bruxer (Figure 3) and the non-bruxer (Figure 4) groups. The bruxer group had 96.7% excellent and 3.3% satisfactory surfaces (due to porcelain chipping and zirconia fracture); 35% excellent color; 87.6% excellent shape (due to three irreparable fractured teeth [0.9%] and five reparable open proximal contacts [1.5%] with another 10%, including the six open proximal contacts without food impaction, were evaluated as having a satisfactory shape); 45% marginal integrity rated as satisfactory (due to over contouring or open margins that led to two cases of secondary caries). The non-bruxer group comprised 98.4% excellent and 1.6% satisfactory surfaces (due to porcelain chipping and zirconia fracture); 45% excellent color; 90.2% excellent shape (due to seven reparable open proximal contacts [2.3%] with another 7.5%, including the six open proximal contacts, without food impaction, evaluated as having a satisfactory shape); and 40% marginal integrity rated as satisfactory (due to over contouring or open margins that led to three cases of secondary caries). Overall, 97.6% and 97.7% of restorations in the bruxer and the non-bruxer groups, respectively, were evaluated as satisfactory and not in need of repair or remake.

**Figure 5:**
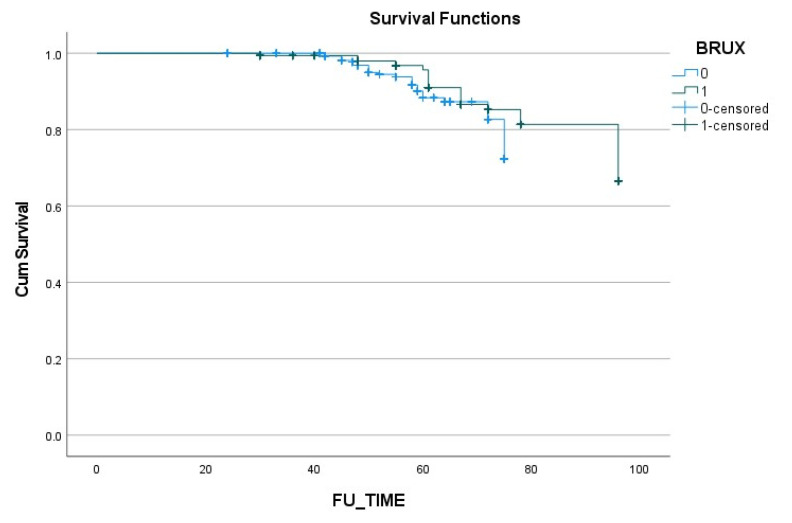
Kaplan–Meier overall survival rate combined with the complications that occurred.

The results of the Kaplan–Meier analysis are presented in Figure 5, and demonstrate similar rates of survival and complications in both groups.

## 4. Discussion

The present study assessed the survival and success rates of monolithic zirconia restorations supported by teeth and implants in bruxer versus non-bruxer patients. There is currently little information regarding this issue since bruxer patients are often excluded from studies evaluating the long-term outcome of restorations. Our results did not reveal any significant differences in the overall survival and success rate of the monolithic zirconia restorations between bruxer and non-bruxer patients; therefore, the null hypothesis was accepted. Nevertheless, some differences between the groups were noted.

The current study compared 15 bruxers with 331 monolithic zirconia restorations versus 25 non-bruxers with 306 monolithic zirconia restorations. All the bruxer patients were diagnosed with “probable” bruxism according to the criteria of the American Academy of Sleep Medicine [13] following a clinical inspection and a self-report. The zirconia restorations had survival rates of 99.2% and 100% for the bruxer and non-bruxer groups, respectively. These results are in accordance with previous reports of high survival rates of monolithic zirconia restorations in bruxer patients [5,7,9]. Nowadays, in full mouth rehabilitations, a total digital workflow can be done with the aid of a Total Face Approach (TFA) 3D Cephalometry [19]. This enables careful digital planning, according to the skeletal class and the vertical dimension, followed by the monolithic milling production of the zirconia.

In the literature, tooth- and implant-born monolithic zirconia restorations in non-bruxers have presented promising treatment outcomes for single crowns and multiunit fixed partial dentures [10,20]. A summary of systematic reviews on the long-term clinical performance and complications of zirconia-based tooth and implant-supported fixed prosthodontic restorations reported excellent 5-year cumulative survival rates despite a number of biological and technical complications [10].

However, it is reasonable to expect a higher rate of biological and technical complications in heavy grinder patients, who are very often excluded from studies on the outcomes of crowns. In the current study, no significant differences between the bruxer and the non-bruxer groups were detected for overall biological and technical complications. The present results contrast with those of some previous studies. Chrcanovic et al. showed that bruxism was a factor associated with a higher risk of screw loosening, screw fracture, ceramic chipping/fracture, and the loss/fracture of acrylic teeth [21]. In addition, the authors concluded that bruxers had a significantly higher risk of prosthesis failure than non-bruxers. In another retrospective study on 2670 patients, bruxism was among the five factors shown to significantly increase the fracture rate of implants [22]. Koenig et al. demonstrated that 80% of all catastrophic failures occurred in patients with clinical signs of bruxism [23]. In the current study, only 1 out of 68 implants failed in the non-bruxer group versus 2 out of 75 implants in the bruxers. The low rate of implant failures observed in both groups in the present study could be explained by the small sample size of implants, which does not allow us to make any conclusions, even though the patients in the bruxer group exert high occlusal stress which may be the cause of the implant disintegration that occurred in this group. Because of the small sample size of implants in the current study, the difference between the survival of implants with either cemented or screw-retained restorations could not be analyzed. The type of restoration can affect the stability of the peri-implant tissues, regardless of the occlusal load [24].

Among the biologic complications, fractured teeth were the only complication with a trend toward a significant difference between the groups, since the three fractured teeth noted were exclusively in the bruxer group. It is possible that a larger sample size might detect the difference between the groups more accurately. In a systematic review of the clinical performance of tooth- or implant-supported zirconia-based fixed partial dentures, only 7 abutment tooth fractures were reported in a total of 27 studies [25]. Our hypothesis to explain this high incidence of complication is that in cases of high occlusal stress, the stiff monolithic zirconia restoration, which is unable to absorb stresses, transmits the stress to the tooth. As a consequence, the tooth fractures at the weakest point (below the covered surface of the crown). Therefore, in bruxer patients, it may be advisable to consider connecting two abutment teeth with minimal tooth structure in order to distribute the high occlusal forces.

Porcelain chipping on the incisal edge of the veneered monolithic restorations was the only technical complication noted in the current study with a significant difference between the groups. This is in agreement with other studies. A 9-year retrospective study that evaluated the survival and success rates of zirconia-based restorations concluded that veneer chipping was significantly influenced by the presence of parafunctional activity [23]. Another study that assessed the up-to-7-year clinical outcomes of implant- and tooth-supported zirconia-based single crowns found that the rate of porcelain chipping was significantly higher in patients with bruxism [26]. Chrcanovic et al. reported that a group of bruxer patients experienced a higher prevalence of mechanical complications than non-bruxers, including a higher rate of fractured porcelain or acrylic teeth, and screw loosening [4]. Similarly, Levartovsky et al. reported a common technical complication of minor veneer porcelain chipping (13.9%) among bruxers [5]. As in the current study, this porcelain chipping could be resolved by polishing. However, in contrast, a consecutive case series reported by Moscovitch described a 100% success rate of monolithic zirconia restorations with or without a labially veneered feldspathic ceramic component [27]. The difference may be attributed to unpresented data concerning parafunctional occlusal activity among the patients in the aforementioned study. We now conclude that it may be preferable to use fully monolithic zirconia restorations, without the veneered feldspathic porcelain, in bruxer patients, because high occlusal loads may induce chipping as a consequence of phase transformation around microcracks in the outer surface of the restoration [28].

Another significant technical complication that was noted in the current study, albeit without a statistical difference between the groups, was the opening of proximal contacts between implant-supported prostheses and adjacent teeth. This result is in accordance with other studies without reference to bruxism. In a systematic review and meta-analysis, the odds ratio of developing open proximal contacts between implant-supported prostheses and adjacent teeth was found to be 2.46 compared to tooth-supported prostheses [29]. Another review of the literature reported open proximal contacts in 34–66% of cases after the insertion of an implant restoration, which was explained by the exertion of force vectors on the teeth while the implant is ankylosed to the bone [30]. Recently, a systematic review and meta-analysis reported a cumulative 41% rate of loss of proximal contacts [31]. Further technical complications such as screw loosening, minor zirconia fracture, and decementation occurred rarely in both groups.

The limitations of the present study include the retrospective nature of the study, the small sample size of patients, and the short follow-up period. In addition, the diagnosis of bruxism was based only on clinical inspection and a self-report without examination by polysomnography. Further well-designed studies with a larger sample size and longer follow-up period are warranted.

## 5. Conclusions

Within the limitations of this retrospective study, we conclude the following:

(1) There were no significant differences in the overall complications and survival rate between bruxer and non-bruxer patients, even though most of the bruxers used an occlusal guard during sleep.
(2) Porcelain chipping on the incisal edge of the veneered monolithic restorations was significantly higher in the bruxer group than in the non-bruxers (*p* = 0.045).
(3) The only biologic complications that exhibited a borderline, although not significant, difference were three fractured teeth exclusively in the bruxer group (*p* = 0.051), which were replaced with implant-supported restorations.

## Figures and Tables

**Table 1:** Number of restorations and abutments in both groups.

Patient Group	Sum of Rest	Veneered Rest	Non-Veneered Rest	Teeth No.	Implants				
					Total Cemented Screwed Maxilla Mandible				
Bruxer	331	95	236	250	75	28	47	27	48
Non-bruxer	306	84	222	232	68	23	39	20	42

Rest = Restoration.

**Table 2:** Student’s *t*-test for mean age, follow-up time, complication, and survival rate.

	Patient Group	N	Mean	Std. Deviation	Std. Error Mean	*p*
Age	Non-bruxer	25	66.68	7.03	1.41	0.157
	Bruxer	15	61.20	13.32	3.44	
Time	Non-bruxer	25	54.12	12.48	2.50	0.327
	Bruxer	15	58.73	16.76	4.33	
Complication	Non-bruxer	25	9.54	11.52	2.30	0.412
	Bruxer	15	14.30	24.73	6.38	
Survival	Non-bruxer	25	100.00	0.00	0.00	0.169
	Bruxer	15	99.22	2.08	0.54	

Time = Follow-Up in Months.

**Table 3:** Biological and technical complications in both groups.

Patient Group		
	Bruxer	Non-Bruxer
Biological		
Secondary caries	2	3
Implant failure	2	1
Fractured tooth	3	0
Technical		
Porcelain chipping	10	3
Loosening of a screw	2	4
Zirconia fracture (minor)	1	2
Loss of retention	1	1
Open proximal contact	11	13
Total number of complications	31	27

**Table 4:** Life table analysis on implant survival level in the bruxer group.

Interval (Years)	No. of Implants	No. of Failures	Interval Survival Rate (%)	Cumulative Survival Rate (%)
0–1 year	75	0	100	100
1–2 years	75	1	98.6	98.6
2–3 years	74	0	100	98.6
3–4 years	74	0	100	98.6
4–5 years	70	0	100	98.6
5–6 years	51	1	98.0	97.3
6–7 years	23	0	100	97.3
7–8 years	5	0	100	97.3

**Table 5:** Life table analysis on implant survival level in the non-bruxer group.

Interval (Years)	No. of Implants	No. of Failures	Interval Survival Rate (%)	Cumulative Survival Rate (%)
0–1 year	68	0	100	100
1–2 years	68	0	100	100
2–3 years	68	0	100	100
3–4 years	57	0	100	100
4–5 years	45	0	100	100
5–6 years	35	0	100	100
6–7 years	18	1	94.4	98.5
7–8 years	10	0	100	98.5

## Data Availability

Not applicable.
